# Bioequivalence study of two subcutaneous formulations of dalteparin: randomized, single-dose, two-sequence, two-period, cross-over study in healthy volunteers

**DOI:** 10.3109/21556660.2013.781504

**Published:** 2013-03-04

**Authors:** C. Gadiko, S. K. Tippabhotla, S. Thota, M. Nakkawar, R. Cheerla, M.R. Betha, V. Vobalaboina

**Affiliations:** Clinical Pharmacology and Pharmacokinetics, Integrated Product Development, Dr. Reddy’s Laboratories Ltd, Bachupally, HyderabadIndia

**Keywords:** Dalteparin, Bioavailability, Bioequivalence, Subcutaneous

## Abstract

**Objective:**

This study assessed relative bioavailability of a new subcutaneous formulation, test (T) (dalteparin sodium 95000 IU/3.8 mL) with the branded product (R) in healthy subjects to meet the regulatory requirements of bioequivalence in the US.

**Methods:**

This was an open label, randomized, single dose, two-sequence, two-period cross-over study under fasting conditions. A total of 88 healthy adult volunteers were randomized to either of the treatment arms (T or R) separated by a washout period of 7 days. Pharmacodynamic surrogates, namely anti-Xa and anti-IIa activity, heparin clotting assay (heptest), and activated partial thromboplastin time (aPTT) were used as a tool to establish bioequivalence between these two formulations. Blood samples were collected up to 36 h post-dose to characterize the primary pharmacokinetic parameters *A*_max,_ AUC_0–_*_t_*, and AUC_0–∞_ for anti-Xa and anti-IIa and heptest; parameters (Δ*t* )_max_ and AU(Δ*t* ) for aPTT.

**Results:**

For anti-Xa activity, the means (SD) of *A*_max_ (IU/mL) were 1.34 (0.25) [range = 0.59–2.03] and 1.39 (0.35) [range = 0.65–2.69]; AUC_0–_*_t_* (IU•h/mL) values were 11.4 (2.76) [range = 2.89–19.5] and 12.1 (2.87) [range = 2.52–21.30]; AUC_0_*_–__∞_* (IU•h/mL) values were 13.1 (3.59) [range = 3.15–28.2] and 14.5 (4.97) [range = 2.79–36.1] for test and branded formulations, respectively. For anti-IIa activity, the means (SD) of *A*_max_ (IU/mL) were 0.34 (0.12) [range = 0.14–0.72] and 0.34 (0.13) [range = 0.11–0.84]; AUC_0–_*_t_* (IU•h/mL) values were 2.05 (0.72) [range = 0.61–4.69] and 2.11 (0.76) [range = 0.84–4.80]; AUC_0_*_–__∞_* (IU•h/mL) values were 2.47 (0.80) [range = 0.76–6.29] and 2.61 (0.86) [range = 1.31–5.36], for test and branded formulations, respectively. The 90% CI for all the primary pharmacokinetic parameters of all the pharmacodynamic surrogates tested met the regulatory bioequivalence criterion of 80.00–125.00%.

**Conclusion:**

The test product met the US regulatory criteria of bioequivalence relative to the branded product in this single dose bioequivalence study. Study limitations include open-label single dose design.

## Introduction

Dalteparin, an antithrombotic drug, is a low molecular weight heparin (LMWH) obtained from unfractionated heparin^1,2^. LMWHs inhibit blood coagulation by binding to antithrombin III, and the resulting complex inhibits clotting factor Xa to a greater extent and factor IIa (thrombin) to a lesser extent^3^. Thus, when these are administered to humans, they preferentially potentiate the inhibition of factor Xa, while they barely affect the activated partial thromboplastin time (aPTT)^4^.

LMWH offers the advantages over heparin in having greater bioavailability^5^, a longer half-life^6^, resistance to inactivation by platelet factor 4^7^, and a relatively lower number of serious adverse reactions, particularly thrombocytopenia^8^. The primary advantage of greater bioavailability of LMWHs over unfractionated heparin gives LMWHs the provision for subcutaneous administration without the need for routine laboratory monitoring or dosage adjustment^9–11^. LMWHs have been used as a first line drug since the time of their introduction for the treatment and prophylaxis of deep vein thrombosis^12^.

Dalteparin enhances the inhibition of factor Xa predominantly and factor IIa to a lesser extent by binding to antithrombin III. It also induces a dose-dependent release of tissue factor pathway inhibitor (TFPI) from the endothelial surface, thus inhibiting the extrinsic pathway in a coagulation cascade. All these effects are directly linked to its anticoagulant property. Due to the high anti-Xa activity of dalteparin, anti-Xa assays are commonly used to monitor responses to treatment and it’s *in vitro* potency^13,14^, but this alone may not accurately reflect the anticoagulant action, because dalteparin also inhibit factor IIa. Hence, for bioequivalence studies, anti-IIa assay data may serve as supportive information of comparable therapeutic outcome. Additionally, heparin clotting assay (heptest) that is sensitive to both anti-Xa and anti-IIa activity, as well as dalteparin-stimulated release of TFPI^15^ and aPTT, serve as pharmacodynamic surrogates in comparative bioavailability studies.

Fragmin (Eisai Inc., Woodcliff Lake, NJ) injection is a branded formulation of dalteparin sodium that is being marketed in the US^16^. A generic version of dalteparin sodium subcutaneous (sc) injection is being developed for marketing in the US. There is no published data on the pharmacokinetics or comparative bioavailability study on dalteparin. Hence, the present study has been designed and conducted to determine the bioequivalence of generic and branded formulations of dalteparin sodium 95,000 IU/3.8 mL sc injection in healthy, adult volunteers under fasting condition for the purpose of marketing the generic formulation in the US.

## Subjects and methods

### Subjects

Healthy male and female volunteers aged between 18–45 years and body mass index between 18.6–29.9 kg/m^2^ were enrolled. The screening procedure was conducted within 28 days prior to the study enrollment (day 1 of the study). All the study participants provided written informed consent prior to the screening procedure. Female subjects were non-pregnant, non-lactating, surgically sterile, and willing to use a suitable and effective double barrier contraceptive method or intra-uterine device from screening through 6 weeks after study completion.

Volunteers were excluded from the study in the following cases: volunteers with a recent history of surgery, active or clinically suspected bleeding diathesis or platelet defects, history or presence of any disease or disorder known to influence bone metabolism, compromise the haemopoietic, renal, hepatic, pulmonary, central nervous, cardiovascular, immunological, gastrointestinal, or any other body system, or any drug allergy or hypersensitivity to heparin, pork, or drug-related products. Volunteer exclusion also includes smokers, consumption of any other drug product, donated blood, those who had been on an abnormal diet, or had substantial changes in their eating habits in the last 30 days prior to study initiation.

### Study design

This was an open-label, randomized, single-dose, two-sequence, two-period, cross-over study under fasting conditions with pharmacodynamic end-points. The study was carried out in accordance with the in-house standard operating procedures, ICH (International Conference on Harmonization) E6 ‘Guideline for Good Clinical Practice’ and the principles enunciated in the Declaration of Helsinki (revised version of Seoul, Korea, 2008). The study was conducted after prior approval of study protocol and study-related documents by the Bio-Kinetic Clinical Applications (1816 west Mt. Vernon, Springfield, MO) Institutional Review Board (IRB).

### Study drugs and study restrictions

Subjects willing to participate provided their written informed consent prior to dosing in period-I. They were randomized to either of the treatments, test (dalteparin sodium injection 95,000 IU/3.8 mL, batch number. JE101, expiration date March 2013) or branded drug (lot number Y03270, expiration date February 2014) using SAS Version 9.1.3. After an overnight fast of at least 10 h, all the subjects received a single subcutaneous dose of 120 IU/kg from the label potency (95,000 IU/3.8 mL) in a U-shaped area around the navel or the upper outer side of the thigh or the upper outer quadrangle of the buttock as per the randomization schedule in each period separated by a washout period of 7 days. For the subjects weighing greater than 83.3 kg, an upper limit of 10,000 IU dose was to be administered. In this study dosage did not exceeded 10,000 IU for any of the subjects. Subjects were instructed to abstain from consuming any alcoholic products, xanthine containing foods and/or beverages (like chocolate, tea, coffee, cola drinks), grapefruit juice, smoking, or chewing tobacco-containing products from 24 h prior to dosing until the last sample collection in each study period. They were also instructed to avoid using any medicine for at least 30 days prior to first study drug administration and until study completion. Urine scan for drugs of abuse and a breath alcohol test were carried out prior to check-in of period-I. Drinking water was restricted from 1 h pre-dose until 1 h post-dose. Drinking water was allowed ad libitum at all other times. Subjects were instructed to remain sitting or in a semi-inclined position for up to 2 h post-dose.

### Efficacy assessment

#### Blood sampling schedule

A total of 19 blood samples were collected from each subject in each period. The venous blood samples were withdrawn at pre-dose (0.0) and at 0.5, 1, 2, 3, 3.5, 4, 4.5, 5, 5.5, 6, 7, 8, 10, 12, 14, 16, 24, and 36 h following drug administration in each period into 3.2% sodium citrate blood collection tubes for anti-Xa, anti-IIa, and aPTT determination and 3.2% trisodium citrate anticoagulant sample tubes for heptest determination. Samples were centrifuged at a 1500 relative centrifugal force at 2–8°C for 15 min. Plasma was separated and stored at −70°C or colder until analysis.

#### Bioassay of anti-Xa, anti-IIa, heptest, and aPTT

Assay methods used for measuring anti-Xa, anti-IIa, heptest, and aPTT were validated according to international guidelines. Calibration curve was established using a 9-point calibration curve and five quality control concentrations were used during assay of samples. The analysts performing the assay of anti-Xa, anti-IIa, heptest and aPTT were kept blinded about the sequence of administration of the treatments to the individual subjects during the entire study period.

### Assessment of anti-Xa and anti-IIa activity

#### Anti-Xa activity

In this activity assay, standards, quality control samples, or test samples were combined with factor Xa and warmed substrate buffer in microtiter tubes. These tubes were incubated at room temperature to allow the activity reaction to progress. Following the incubation, the reaction was stopped by the addition of 20% glacial acetic acid. The optical density (OD) of the plate is read at 405 nm and the color intensity was inversely proportional to the quantity of dalteparin sodium present. The dalteparin sodium concentration results in µg/mL were converted into IU/mL for anti-Xa activity, using a conversion factor of 0.140: as 1 µg/mL is equivalent to 0.140 IU/mL anti-Xa activity. The linearity of the assay method range from 1.00–12.00 µg/mL, with a limit of quantitation of 1.00 µg/mL (equivalent to 0.140 IU/mL of dalteparin). Intra-day and inter-day precision ranges from 4.5–8.9% and 4.6–13.6%, while the intra-day and inter-day accuracy ranges from 4.1–11.8% and −1.1–9.6%, respectively.

#### Anti-IIa activity

In this activity assay, dalteparin is analyzed as a complex in the presence of antithrombin. Thrombin in excess is neutralized in proportion to the amount of dalteparin sodium present, which determines the amount of dalteparin sodium-antithrombin complex formed. The remaining amount of thrombin hydrolyzes a chromogenic substrate, liberating a chromophoric group which emits color at 405 nm. The dalteparin concentration results in µg/mL were converted into IU/mL for anti-IIa activity in IU/mL using a conversion factor of 0.056: as 1 µg/mL is equivalent to 0.056 IU/mL anti-IIa activity. The linearity of the assay method range from 1.00–10.00 µg/mL, with a limit of quantitation of 1.00 µg/mL (equivalent to 0.056 IU/mL of dalteparin). Intra-day and inter-day precision ranges from 10.2–16.9% and 8.6–14.4%, while the intra-day and inter-day accuracy ranges from 1.2–10.2% and −4.4–4.3%, respectively.

### Assessment of heptest and aPTT

Automated coagulation analyzer (STA Compact® instrument) was used for processing heparin clotting and aPTT assays. Assays were based on the ability of the dalteparin product to inhibit primarily factor Xa and also factor IIa activity. The principle in chromogenic assays uses the enzyme and substrate which works on kinetics and instrument measures the rate of change in optical density at 405 nm and at different intervals of time for 1 min. Hence, the dalteparin level in the plasma samples being tested were automatically displayed in IU/ml in the ‘test panel/test status’ screen of the instrument. Intra-day and inter-day precision for heptest ranges from 2.4–4.4% and 1.0–4.7% and for aPTT from 1.1–8.2% and 1.4–2.4%, respectively. Intra-day and inter-day accuracy for heptest ranges from 3.3–11.3% and −3.5–4.2% and for aPTT from 3.8–9.6% and −2.1–5.4%, respectively.

### Pharmacokinetic and statistical analysis

Plasma activities of anti-Xa, anti-IIa, heptest and aPTT were used in all pharmacokinetic calculations. Pharmacokinetic parameters were derived individually for each analyzed subject from the anti-Xa, anti-IIa, heptest activity vs time profiles of dalteparin in plasma and for aPTT from a plot of change in clotting time from baseline vs time profile. Data set for the estimation of pharmacokinetic parameters was prepared by using WinNonlin professional software, version 5.3. Actual time-points were used for the estimation of pharmacokinetic parameters. The pharmacokinetic parameters for anti-Xa, anti-IIa and heptest included maximum measured plasma activity (*A*_max_), area under the plasma activity time curve up to time of the last quantifiable activity (AUC_0–_*_t_*), area under the plasma activity time curve up to time infinity (AUC_0–∞_), time to reach maximum concentration (*T*_max_), terminal phase elimination rate constant (*K_e_*), half-life (*t*_½_), AUC_0–_*_t_*/AUC_0–∞_ and AUC_0–∞_ (anti-Xa)/AUC_0–∞_ (anti-IIa). Pharmacokinetic parameters for aPTT included maximum measured change in clotting time compared to baseline (Δ*t*)_max_, area under the curve for a plot of change in clotting time from baseline vs time AU(Δ*t*), and time to reach maximum measured change in clotting time compared to baseline (*T*_max_).

Statistical analysis of pharmacokinetic data was performed by using SAS Version 9.1.3. Comparison of the pharmacokinetic parameters *A*_max,_ AUC_0–_*_t_*, AUC_0–∞_ for anti-Xa, anti-IIa, heptest; parameters (Δ*t*)_max_, AU(Δ*t*) for aPTT; and AUC_0–_*_∞_* (anti-Xa)/AUC_0–_*_∞_* (anti-IIa) ratio for both un-transformed and ln-transformed data with respect to test and branded formulations was done using ANOVA by means of the General linear model (GLM) procedure. This model served as a basis to study the significance of various effects such as period, sequence, and subject nested within sequence. Statistical inferences were based on log-transformed values for *A*_max_, AUC_0–_*_t_* and AUC_0–_*_∞_* parameters for anti-Xa, anti-IIa, heptest and (Δ*t*)_max_ and AU(Δ*t*) for aPTT. Bioequivalence of the test product with that of the branded product under fasting conditions was concluded if the 90% confidence interval (CI) fell within the acceptance range of 80.00–125.00% for ln-transformed pharmacokinetic parameters *A*_max_, AUC_0–_*_t_* and AUC_0–∞_ for anti-Xa and anti-IIa activities of dalteparin and heptest, Δ*t*_max_ and AU(Δ*t*) for aPTT^17^. In addition to the above parameters, bioequivalence also tested for the point estimate (test/branded) of ln-transformed parameter AUC_0–∞_ (anti-Xa)/AUC_0–∞_ (anti-IIa) ratio, which should be within bioequivalence limits of 80.00–125.00%. *T*_max_ was assessed using the non-parametric test on untransformed data.

A sample size of 88 subjects (including dropouts) was determined to be sufficient to establish bioequivalence between dalteparin formulations under fasting conditions with adequate power considering the following estimates: test (T)/branded (R) ratio ∼116%, intra-subject %CV ∼20%, power ≥80%, significance level (*α*) 5%, and bioequivalence limits 80–125%.

### Safety assessment

Safety was assessed from the screening period to the end of the study. It was assessed through medical history, physical examination, vital signs assessment, ECG, clinical laboratory parameters (e.g., serum chemistry, hematology, serology, and urine analysis including serum pregnancy test for female volunteers) at the time of screening and monitoring of adverse events, vitals measurement, and subjective symptomology during the study. Safety exit procedures including vital sign measurements and hematology, chemistry, and urinalysis lab work were performed prior to each subject’s termination from the clinical trial. In addition, a serum pregnancy test was performed on all female subjects.

## Results

### Study participants

Out of the total 88 enrolled subjects, 83 subjects completed the clinical phase of the study and were included in the final pharmacokinetic and statistical analysis. Two subjects (41 and 44) were prematurely terminated due to non-compliance to protocol requirements, and three subjects (52, 57, and 68) withdrew their consent for further participation in the trial. Among the three subjects, subject 57 received both the treatments, but withdrew consent due to an adverse event of anxiety during period 2. Hence, as per the study protocol, all the collected samples of this subject were analyzed due to safety reasons, but the subject’s data was not considered for pharmacokinetic and statistical analysis. Demographic data of the subjects completing the bioequivalence study are summarized in Table 1.

**Table 1.** Demographic profile of the subjects completing the bioequivalence study (*n* = 83).

Demographic variable	Test product (T)	Branded product (R)
Age (years)		
Arithmetic mean (SD)	30.0 (8.6)	30.0 (8.6)
Range	19–45	19–45
Median	28	28
Sex, *n*		
Male	44	44
Female	39	39
Height (cm)		
Arithmetic mean (SD)	173.6 (10.35)	173.6 (10.35)
Range	155–201	155–201
Median	172	172
Weight (kg)		
Arithmetic mean (SD)	75.8 (12.6)	75.8 (12.6)
Range	50.6–119.6	50.6–119.6
Median	76	76
Race, *n* (%)		
Black	9 (10.8%)	9 (10.8%)
White	72 (86.7%)	72 (86.7%)
American Indian/Alaskan	2 (2.3%)	2 (2.3%)
Body mass index (kg/m^2^)		
Arithmetic mean (SD)	25.1 (3.2)	25.1 (3.2)
Range	18.6–29.9	18.6–29.9
Median	25	25

### Pharmacokinetic parameters of pharmacodynamic surrogates

The pharmacokinetic parameters evaluated for anti-Xa and anti-IIa activities, heptest, and aPTT are tabulated in Table 2. For anti-Xa activity, the mean (SD) *A*_max_ (IU/mL) values were 1.34 (0.25) [range = 0.59–2.03] with the test formulation, and 1.39 (0.35) [range = 0.65–2.69] with the branded formulation. The mean (SD) of AUC_0–_*_t_* (IU•h/mL) values were 11.4 (2.76) [range = 2.89–19.5] with the test formulation, and 12.1 (2.87) [range = 2.52–21.30] with the branded formulation. Corresponding AUC_0_*_–__∞_* (IU•h/mL) values were 13.1 (3.59) [range = 3.15–28.2] and 14.5 (4.97) [range =2.79–36.1]. The median (range) (h) values for *t*_max_ were 4 (2–7) with the test formulation and 4 (2–5.50) with the branded formulation. The Least Square Mean (LSM) ratios (%) (90% CI) of the log-transformed values were 96.6 (93.35–99.95%) for *A*_max_, 93.6 (90.59–96.77%) for AUC_o–_*_t_*, and 91.2 (86.26–96.45%) for AUC_0–∞_, respectively.

**Table 2.** Pharmacokinetic parameters of anti-Xa, anti-IIa, and heparin clotting assay (heptest) after single dose subcutaneous injection of test and branded formulations of dalteparin in healthy subjects (*n* = 83*).

PK parameter	Anti-Xa activity	Anti-IIa activity	heptest
Test (T)	Branded (R)	Test (T)	Branded (R)	Test (T)	Branded (R)
Arithmetic mean (SD)
*A*_max_ (IU/mL)	1.34 (0.25)	1.39 (0.35)	0.34 (0.12)	0.34 (0.13)	0.84 (0.16)	0.84 (0.17)
AUC_0–_*_t_*
(IU•h/mL)	11.4 (2.76)	12.1 (2.87)	2.05 (0.72)	2.11 (0.76)	6.76 (1.32)	7.05 (1.39)
AUC_0–∞_
(IU•h/mL)	13.1 (3.59)	14.5 (4.97)	2.47 (0.80)	2.61 (0.86)	7.36 (1.34)	7.68 (1.38)
AUC_0–_*_t_*/AUC_0–∞_	0.88 (0.08)	0.86 (0.11)	0.83 (0.09)	0.81 (0.10)	0.92 (0.03)	0.92 (0.03)
*T*_max_ (h)^a^	4.00 [2.00–7.00]	4.00 [2.00–5.50]	4.00 [2.00–8.00]	4.00 [2.00–7.00]	3.50 [2.00–6.00]	4.00 [2.00–8.00]
*t*_1/2_ (h)	6.97 (8.47)	10.20 (14.80)	3.11 (1.81)	3.73 (2.56)	3.2 (0.93)	3.34 (0.89)
*K_e_* (h^−1^)	0.16 (0.11)	0.13 (0.09)	0.27 (0.10)	0.24 (0.11)	0.23 (0.06)	0.22 (0.05)

SD, Standard deviation. ^a^*T*_max_ was analyzed using a non-parametric test on untransformed data and reported as median [range]. * *n* = 83; Subjects 41, 44, 52, 57, and 68 had not completed both periods of the study and, hence, were excluded from pharmacokinetic and statistical analysis.

A relatively lower anti-IIa activity was observed for both the treatments. The mean (SD) anti-IIa *A*_max_ (IU/mL) values were 0.34 (0.12) [range = 0.14–0.72] and 0.34 (0.13) [range = 0.11–0.84] for test and branded formulations, respectively. The AUC_0–_*_t_* (IU•h/mL) values were 2.05 (0.72) [range = 0.61–4.69] for test formulation and 2.11 (0.76) [range = 0.84–4.80] for branded formulation. Corresponding AUC_0_*_–__∞_* (IU•h/mL) values were 2.47 (0.80) [range = 0.76–6.29] and 2.61 (0.86) [range = 1.31–5.36] for test and branded formulations, respectively.

For heptest, the LSM ratios (90% CI) for *A*_max_, AUC_0–_*_t_*, and AUC_0–∞_ were 99.2% (96.86–101.55%), 95.9% (94.28–97.51%), and 95.8% (94.32–97.31%), respectively; for aPTT the LSM ratios (90% CI) for the primary pharmacokinetic parameters, (Δ*t*)_max_ and AU(Δ*t*)) were 103% (98.57–106.64%) and 100% (92.46–108.06%).

**Figure 1.**
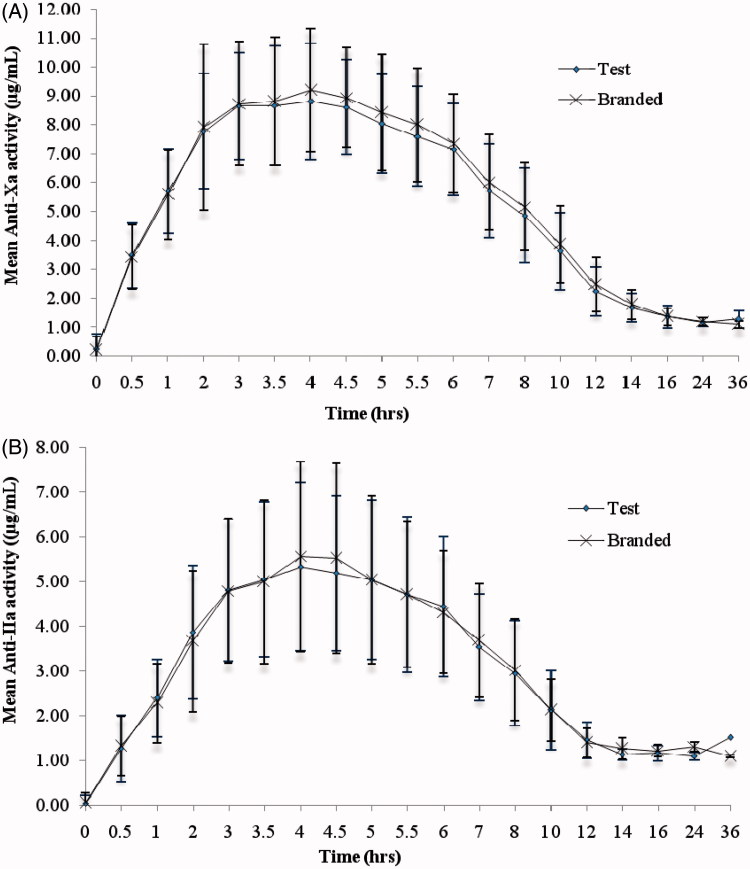
(A) anti-Xa activity and (B) anti-IIa activity vs time curve after single dose administration of test and branded product of dalteparin sodium.

**Figure 2.**
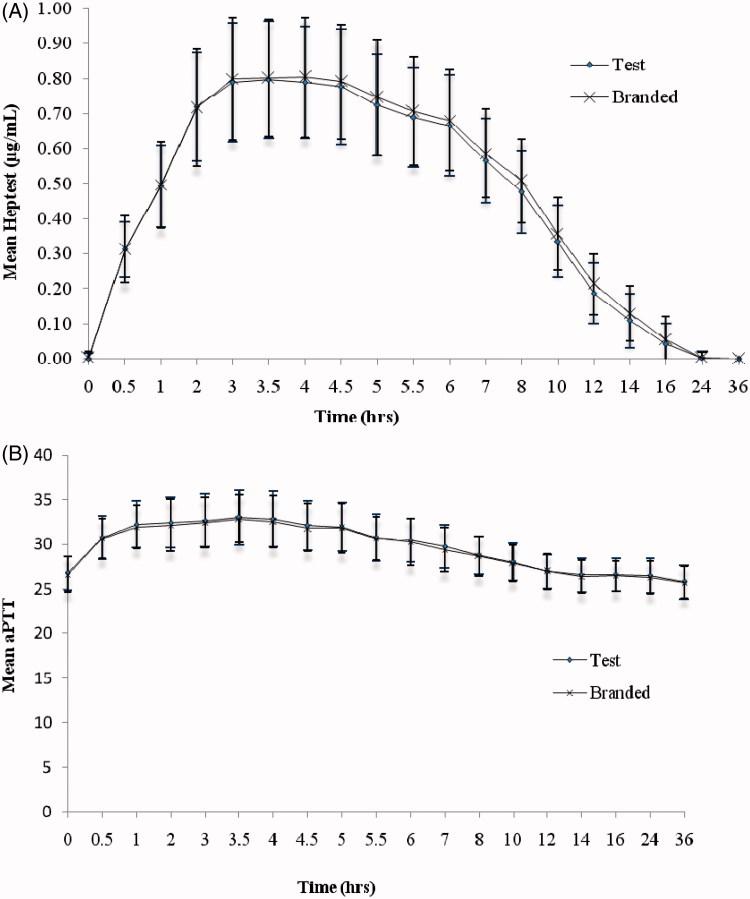
(A) heptest and (B) aPTT activity vs time curve after single dose administration of test and branded product of dalteparin sodium.

The mean (SD) of anti-Xa, anti-IIa activity (Figure 1), heptest and aPTT (Figure 2) versus time profiles of test and branded formulations were superimposed on each other. In comparison to anti-Xa activity, a relatively lower effect in anti-IIa activity was observed for both the test and branded formulations. There were 10 subjects with body weight > 83.3 kg and, hence, their dose is capped at 10,000 IU. The *C*_max_, AUC_0–_*_t_*, and AUC_0–∞_ were relatively lower for these subjects compared to the remaining 73 subjects. However, as both the groups greatly differ in their sizes, the statistical test was not performed on the data. The 90% CI and LSM ratios for the primary pharmacokinetic parameters [*A*_max_, AUC_0–_*_t_* and AUC_0–∞_ of anti-Xa, anti-IIa activities, and heptest; parameters (Δ*t*)_max_, AU(Δ*t*) for aPTT] of all the pharmacodynamic surrogates tested met the regulatory bioequivalence criterion of 80.00–125.00%. The point estimate of AUC_0–∞_ (anti-Xa)/AUC_0–∞_ (anti-IIa) lies within the 90% CI of 80.00–125.00% (Tables 3 and 4). ANOVA applied on log-transformed values for *A*_max_, AUC_0–_*_t_*, and AUC_0–∞_ parameters for anti-Xa, anti-IIa, and heptest, (Δ*t*)_max_, and AU(Δ*t*) for aPTT for the difference between all the factors, sequence, subject within sequence, and period were found to be statistically insignificant (*p* < 0.05), indicative of an absence of significant differences between the test and branded formulations.

**Table 3.** Pharmacokinetic parameters of activated partial thromboplastin time (aPTT) after single dose subcutaneous injection of test and branded formulations of dalteparin (*n* = 83*).

PK parameter	Test product (T), Mean (SD)	Branded product (R), Mean (SD)
(Δ*t*)_max_ (s)	6.81 (1.84)	6.63 (1.99)
AUC(Δ*t*) (s•h)	43.7 (19.7)	43.5 (17.3)
*T*_max_ (h)^a^	3.00 [1.00–5.00]	3.00 [0.50–5.00]

SD, Standard deviation. ^a^Values reported as median [range]. *n = 83; Subjects 41, 44, 52, 57, and 68 had not completed both periods of the study and, hence, were excluded from pharmacokinetic and statistical analysis.

**Table 4.** Summary statistics of bioequivalence study data after single dose subcutaneous injection of test and branded formulations of dalteparin (*n* = 83*).

Parameter^a^	Geometric least square means			
	Test (T)	Branded (R)	% (T/R) ratio	90% CI	% power
*Anti-Xa activity*
*A*_max_ (IU/mL)	1.31	1.36	96.6	93.35–99.95	100.0
AUC_0–_*_t_* (IU•h/mL)	11	11.7	93.6	90.59–96.77	100.0
AUC_0–∞_ (IU•h/mL)	12.5	13.7	91.2	86.26–96.45	99.9
*Anti-IIa activity*
*A*_max_ (IU/mL)	0.32	0.32	99.9	94.68–105.45	99.9
AUC_0–_*_t_* (IU•h/mL)	1.94	1.99	97.3	92.53–102.41	99.9
AUC_0–∞_ (IU•h/mL)	2.36	2.48	94.9	90.31–99.79	99.9
*Heptest*
*A*_max_ (IU/mL)	0.82	0.83	99.2	96.86–101.55	100.0
AUC_0–_*_t_* (IU•h/mL)	6.63	6.92	95.9	94.28–97.51	100.0
AUC_0–∞_ (IU•h/mL)	7.24	7.56	95.8	94.32–97.31	100.0
*aPTT*
(Δ*t*)_max_ (s)	6.81	6.63	103	98.57–106.64	100.0
AU(Δ*t*) (s.h)	43.7	43.6	100	92.46–108.06	99.9
*AUC_0–__∞_ (anti-Xa)/AUC_0–__∞_ (anti-IIa) ratio*
AUC_0–∞_ (anti-Xa)/AUC_0–∞_ (anti-IIa)	5.31	5.53	96.1	89.48–103.19	

^a^Nature log-transformed. *n = 83; Subjects 41, 44, 52, 57, and 68 had not completed both periods of the study and, hence, were excluded from pharmacokinetic and statistical analysis.

### Safety results

There were no clinically significant changes in any laboratory or vital sign parameters noted for any subject throughout the clinical study, with the exception of one subject who experienced clinically significant changes in his ALT (alanine amino transferase) and AST (aspartate amino transferase). A total of 37 adverse events (AEs) were reported by 19 subjects during the conduct of the study. The incidence of adverse events observed for test and branded formulation against the body system are presented in Table 5. Out of the 37 AEs, 26 AEs occurred in subjects receiving the test formulation and 10 AEs in subjects receiving the branded formulation. One adverse event, nausea, occurred prior to administration of treatment in period-I and, hence, it is not related to any of the treatments administered. The AEs were mild or moderate in intensity, and the investigator deemed all the AEs to have no reasonably possible relationship to the study drug. The relationship to the study treatment characterized as ‘No reasonable possibility’ applies to those adverse events which, after careful consideration, are clearly due to extraneous causes (disease, environment, etc.) or to those adverse events for which, after careful medical consideration at the time they are evaluated, are judged to be unrelated to the test drug. All the observed adverse events were considered to be resolved. Given this information, the study drug appeared to be well-tolerated and there were no safety concerns observed.

**Table 5.** Incidence of adverse events observed for test and branded formulation of dalteparin in this bioequivalence study (*n* = 88).

Body system/Adverse event	Test product (T)	Branded product (R)
*Body as a whole*		
Lightheaded	1	0
Headache	7	4
Diaphoretic	1	0
Elevated ALT	1	0
Elevated AST	1	0
Anemia	1	0
Anxiety	0	1
Chills	1	0
*Gastrointestinal*		
Nausea	3	0
Diarrhea	1	0
Anorexia	1	0
*Respiratory*		
Rhinorrhea	1	0
Cough	1	0
*Abdomen*		
Bruising at injection site	1	2
Burning sensation at injection site	1	2
Pain at injection site	1	0
Tenderness at injection site	0	1
Upper right quadrant discomfort	1	0
Tenderness upper right quadrant	1	0
Abdominal pain upper right quadrant	1	0
Total	26	10

## Discussion

The biological origin of dalteparin necessitates that the pharmacokinetic as well as bioavailability studies are carried out using pharmacodynamic surrogates. Validated bioassays were used to determine anti-Xa and anti-IIa activities, heptest, and aPTT. The pharmacodynamic surrogates used in this study are based on the previously published article on bioequivalence testing of enoxaparin^18^. From *in vitro* studies, it is demonstrated that, at high concentrations, heptest provides a measure of both anti-Xa and anti-IIa activities of LMWH, whereas, at lower concentrations, this test predominantly measure anti-Xa activity. Thus, the sensitivity of heptest in measuring anti-IIa activity is significantly lower than for anti-Xa activity^19^. Recent chromogenic assays, namely Extrinsic Coagulation Activity Assay (EXCA) or Intrinsic Coagulation Activity Assay (INCA), were the two prominent ultra-sensitive assays used globally for determination of thrombin activity in plasma^20^. These new factor IIa and/or factor Xa generation assays (such as the EXCA or the INCA) would be superior to just the determination of anti-Xa or heptest. A statistical test used to compare the primary pharmacokinetic parameters (*A*_max_, AUC_0–_*_t_*, and AUC_0–∞_) for anti-Xa, anti-IIa activity, and heptest; and the parameters (Δ*t*)_max_ and AU(Δ*t*) for aPTT did not show statistically significant differences between the two dalteparin formulations. Also, a relatively lower anti-IIa activity is observed for both the test and branded formulations indicative of its predominant anti-Xa activity and only a substantial inhibition of factor IIa. The power of test for Ln-transformed pharmacokinetic parameters, *A*_max_, AUC_0–_*_t_*, and AUC_0–∞_ for all the four pharmacodynamic surrogates was >80.0%, even after removal of five subjects from PK and statistical analysis. The difference in mean half-life value observed between test and branded formulation is due to the higher values (>20 h) reported for four subjects (64, 66, 71, and 87) on receipt of test formulation and in six subjects (3, 4, 12, 19, 67, and 73) upon receipt of branded formulation. The higher half-life observed in these subjects indicates slower removal of drug from plasma, thereby producing a prolonged/sustained effect. This was also supported by the low K_el_ values observed in these subjects. Removal of these subjects in the half-life calculation resulted in similar half-lives between both the formulations. The revised half-lives (mean ± SD) observed for test (*n* = 79) and branded (*n* = 77) formulations were 5.38 ± 2.96 and 7.42 ± 9.67, respectively, which were not statistically significant (*p* > 0.05). The 90% confidence intervals for the ratio of geometric LSM of the primary pharmacokinetic parameters and the point estimate of AUC_0–∞_ (anti-Xa)/AUC_0–∞_ (anti-IIa) ratio lie within the regulatory acceptance interval of 80.00–125.00%, thus permitting one to conclude for bioequivalence.

The limitations of the present bioequivalence study were a single dose, open-label design and use of less sensitive assay methods like heptest rather than the newer EXCA or INCA for measuring anti-IIa and/or anti-Xa activity. This was an open-label study where the assessment of adverse events was not blinded.

## Conclusion

The results of this single dose bioequivalence study indicate that the test and branded formulations of dalteparin sodium injection met the US regulatory set criteria of bioequivalence in healthy subjects under fasting conditions.

## Transparency

### Declaration of funding

This study was sponsored by Dr Reddy’s Laboratories Pvt. Ltd, Hyderabad, India.

### Declaration of financial/other relationships

All the authors were employees of Dr Reddy’s Laboratories Limited, Hyderabad, and they have reported to have no conflicts of interest regarding the content of this article. The authors have received no payment for writing or publishing the manuscript.

## Acknowledgments

The authors thank all the clinical site staff and the study subjects for their contribution to this study. Also the authors wish to thank the management of Dr Reddy’s Laboratories Limited, Hyderabad for allowing this work to be published.
